# Structural basis for the inhibition of human angiotensin‐1 converting enzyme by fosinoprilat

**DOI:** 10.1111/febs.16543

**Published:** 2022-06-16

**Authors:** Gyles E. Cozier, Emma C. Newby, Sylva L. U. Schwager, R. Elwyn Isaac, Edward D. Sturrock, K. Ravi Acharya

**Affiliations:** ^1^ Department of Biology and Biochemistry University of Bath UK; ^2^ Department of Integrative Biomedical Sciences, Institute of Infectious Disease and Molecular Medicine University of Cape Town South Africa; ^3^ School of Biology University of Leeds UK

**Keywords:** angiotensin‐1‐converting enzyme, domain selectivity, enzyme structure, inhibitor binding, metalloprotease, X‐ray crystallography

## Abstract

Human angiotensin I‐converting enzyme (ACE) has two isoforms, somatic ACE (sACE) and testis ACE (tACE). The functions of sACE are widespread, with its involvement in blood pressure regulation most extensively studied. sACE is composed of an N‐domain (nACE) and a C‐domain (cACE), both catalytically active but have significant structural differences, resulting in different substrate specificities. Even though ACE inhibitors are used clinically, they need much improvement because of serious side effects seen in patients (~ 25–30%) with long‐term treatment due to nonselective inhibition of nACE and cACE. Investigation into the distinguishing structural features of each domain is therefore of vital importance for the development of domain‐specific inhibitors with minimal side effects. Here, we report kinetic data and high‐resolution crystal structures of both nACE (1.75 Å) and cACE (1.85 Å) in complex with fosinoprilat, a clinically used inhibitor. These structures allowed detailed analysis of the molecular features conferring domain selectivity by fosinoprilat. Particularly, altered hydrophobic interactions were observed to be a contributing factor. These experimental data contribute to improved understanding of the structural features that dictate ACE inhibitor domain selectivity, allowing further progress towards designing novel 2nd‐generation domain‐specific potent ACE inhibitors suitable for clinical administration, with a variety of potential future therapeutic benefits.

**Database:**

The atomic coordinates and structure factors for nACE‐fosinoprilat and cACE‐fosinoprilat structures have been deposited with codes 7Z6Z and 7Z70, respectively, in the RCSB Protein Data Bank, www.pdb.org.

## Graphical abstract

Analysis of high‐resolution crystal structures of angiotensin I‐converting enzyme (ACE) N‐ and C‐domains in complex with fosinoprilat provide further insights into the requirements for high‐affinity domain‐specific inhibition that is required to reduce side effects of treatments that target ACE. In addition, the data highlights where modifications or redesign of fosinoprilat are needed to develop novel insecticides that act via ACE analogues.

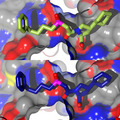

**Abbreviations**
ACE: angiotensin‐1‐converting enzyme
Ang I: angiotensin I
Ang II: angiotensin II
AnoACE2: angiotensin‐1‐converting enzyme homologue from *Anopheles gambiae*
cACE: angiotensin‐1‐converting enzyme C‐domain
nACE: angiotensin‐1‐converting enzyme N‐domain
RAAS: renin–angiotensin–aldosterone system
sACE: somatic angiotensin‐1‐converting enzyme
tACE: testis angiotensin‐1‐converting enzyme

## Introduction

Human angiotensin‐1‐converting enzyme (ACE, EC3.4.15.1) is a key zinc‐dependent dipeptidyl carboxypeptidase with two human isoforms: somatic ACE (sACE, found in somatic tissues) and testis ACE (tACE, found in germinal cells) [1, 2]. sACE is widely expressed and was identified through its role in the renin–angiotensin–aldosterone system (RAAS) where it hydrolyses angiotensin I to active angiotensin II [3]. It is a type I transmembrane glycoprotein, with a short C‐terminal cytosolic domain, a hydrophobic plasma membrane anchoring sequence and an extensively glycosylated (30% glycosylation) extracellular domain comprising of two catalytically active domains (nACE and cACE) [4, 5]. nACE and cACE share a sequence identity of 60% overall and 89% at the active site [2] and their structures as individual domains have been elucidated [6, 7]. They show a conserved overall shape each divided into two subdomains with a flexible N‐terminal lid and central catalytic zinc ion coordinated by the HEXXH motif, where X is any amino acid [4, 8].

Angiotensin‐1‐converting enzyme has numerous substrates in addition to angiotensin I, including substance P, *N*‐acetyl‐seryl‐aspartyl‐lysyl‐proline (Ac‐SDKP), enkephalins, luteinising hormone‐releasing hormone (LHRH), gonadotropin‐releasing hormone (GnRH), kinins, neurotensin, *N*‐formylmethionine‐leucyl‐phenylalanine and amyloid‐beta [9, 10, 11, 12, 13, 14, 15, 16]. Consequently, sACE is a promiscuous enzyme implicated in a range of physiological processes such as blood pressure control, fibrosis, immunomodulation, renal development and function, haematopoiesis, myelopoiesis, erythropoiesis, amyloid‐beta clearance and cell signalling [17, 18, 19, 20, 21, 22, 23, 24, 25, 26].

Further detailed analysis of the individual active sites with bound inhibitors, substrates and peptides have provided specific details of the broad substrate specificity of sACE, with the S_1_ and S_2_′ pockets of cACE being more lipophilic and forming more favourable interactions with hydrophobic substrates, compared to the more polar nACE active site [27]. These differences between the two domains manifest in their physiological functions, whereby cACE is mainly responsible for angiotensin I cleavage [5, 28]; nACE primarily cleaves Ac‐SDKP, GnRH and Aβ [11, 12, 16, 29, 30]; and both domains process bradykinin equally [30]. Therapeutically, this presents the challenge of domain‐specific inhibition of ACE to control definitive physiological functions, requiring a detailed understanding of the structural features that facilitate them. This is highlighted by the serious side effects seen in patients (~ 25–30%) with long‐term treatment by ACE inhibitors that are nonselective for nACE or cACE [31]. Most notably, total inhibition of bradykinin cleavage commonly results in a cough, but more seriously can cause angioedema, which can prove fatal [32]. Therefore, selective inhibition of cACE would treat hypertension while minimising adverse effects. Likewise, selective N‐domain inhibition facilitates Ac‐SDKP accumulation, which has anti‐inflammatory and antifibrotic effects and thus could be used therapeutically to treat fibrosis. Furthermore, recent findings demonstrating the involvement of cACE in modulating immune cell metabolic activity and function suggest a potential application of domain‐specific inhibition in treating autoimmune disorders [33].

**Fig. 1:**
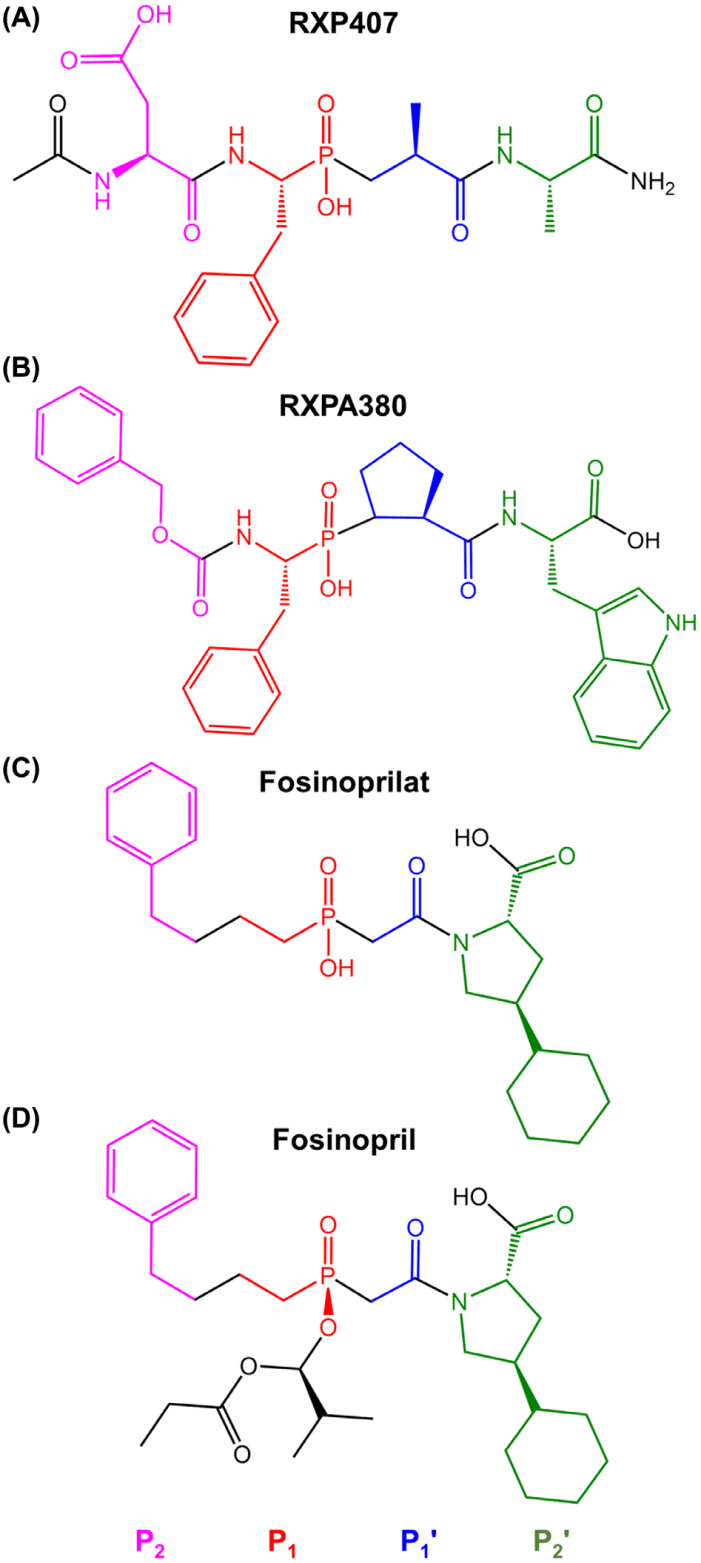
Chemical structures of ACE inhibitors. Schechter–Berger nomenclature is used to indicate the inhibitor moieties occupying the nonprime and prime subsites either side of the zinc binding phosphinic group.

Currently, several domain‐selective ACE inhibitors exist, in particular phosphinic compounds RXP407, which has approximately 200‐fold selectivity for nACE [33, 34] and RXPA380, whose *K*_i_ is 3000‐fold lower for cACE than nACE [35] (Fig. 1A,B). Theoretically, these compounds would be ideal for the treatment of fibrosis and hypertension, respectively; however, their large size and low bioavailability make them poor drug candidates [2, 36].

Fosinopril (Monopril®), administered as a prodrug, undergoes both hepatic and renal elimination, making it suitable for treating hypertension in patients with kidney dysfunction [37]. Fosinoprilat, the hydrolysed form of the drug, is highly lipophilic, composed of a phenyl group joined by a four‐carbon chain (P_2_ and P_1_) to the zinc binding phosphinic acid moiety, a P_2_′‐P_1_′ peptide backbone mimic and a P_2_′ pyrrolidine ring with carboxy‐terminal mimic and cyclohexane substituents (Fig. 1C). Fosinopril is esterified on the phosphinate group of fosinoprilat (Fig. 1D).

Regarding the domain specificity of fosinoprilat, binding assays have been carried out with the His‐His‐Leu (HHL) peptide and Ac‐SDKP as C‐domain and N‐domain‐specific substrates, respectively. The *K*_i_ for the inhibition of HHL hydrolysis was 0.29 nm, and for Ac‐SDKP, hydrolysis was 0.06 nm [38], indicating almost 5‐fold N‐domain selectivity. This assay was repeated using acetyl‐seryl‐aspartyl‐(*N*‐acetyl)‐lysyl‐proline (AcSDAcKP), a novel N‐domain‐specific substrate, yielding a *K*_i_ of 0.13 nm [39]. These data suggest fosinoprilat has a tight binding affinity for both the N‐ and C‐catalytic sites, and a low level of nACE selectivity.

In order to understand the binding mode of fosinoprilat with nACE and cACE, we have now determined high‐resolution crystal structures of nACE and cACE in complex with fosinoprilat at 1.75 and 1.85 Å, respectively. These structural features should be useful in the design of potent domain‐selective inhibitors of ACE, like RXP407 and RXPA380, but retaining the bioavailability of fosinoprilat.

## Results

### Inhibition characterisation of fosinoprilat

Fixed‐time fluorimetric assays using the substrate Z‐Phe‐His‐Leu [31] were performed to determine the selectivity of fosinoprilat for the two ACE domains. To quantitatively assess the selectivity, fosinoprilat inhibition was further characterised on the basis of inhibitory binding constants (*K*_i_). Fosinoprilat displayed low nanomolar inhibition of nACE (*K*_i_ = 4.11 ± 0.38 nm) and cACE (*K*_i_ = 0.15 ± 0.01 nm). The inhibition of cACE by fosinoprilat was notably higher than that of nACE with a 27.4‐fold cACE selectivity. This is in contrast to the low‐level nACE selectivity observed in the sACE assays using domain‐specific substrates described above. This difference might have resulted from the use of substrates in the sACE assays that were not 100% domain‐specific, or the synergistic effects of the two domains in sACE. In general, for dissecting out the active site interactions that are responsible for driving domain selectivity we have found the truncated domains more helpful. However, it is important that the interdomain cooperativity for the binding of certain inhibitors is also taken into consideration [40, 41, 42, 43].

### Overall fosinoprilat complex structures

**Fig. 2:**
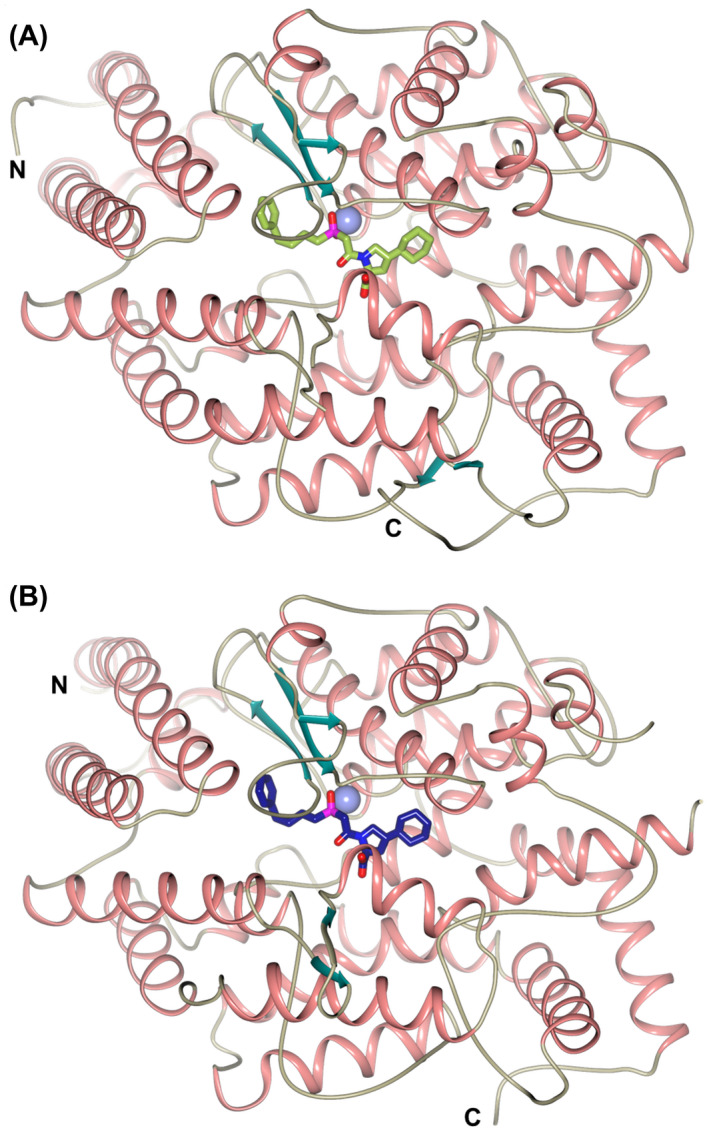
Schematic representation of the overall structure of nACE‐ and cACE‐fosinoprilat complexes. (A) nACE‐fosinoprilat, (B) cACE‐fosinoprilat. Fosinoprilat molecules are shown as green (nACE) and blue (cACE) sticks. Zinc ions are depicted as lilac spheres, with helices, β‐strands and loops coloured in rose, dark cyan and tan, respectively. Figure was generated using ccp4mg [66].

**Fig. 3:**
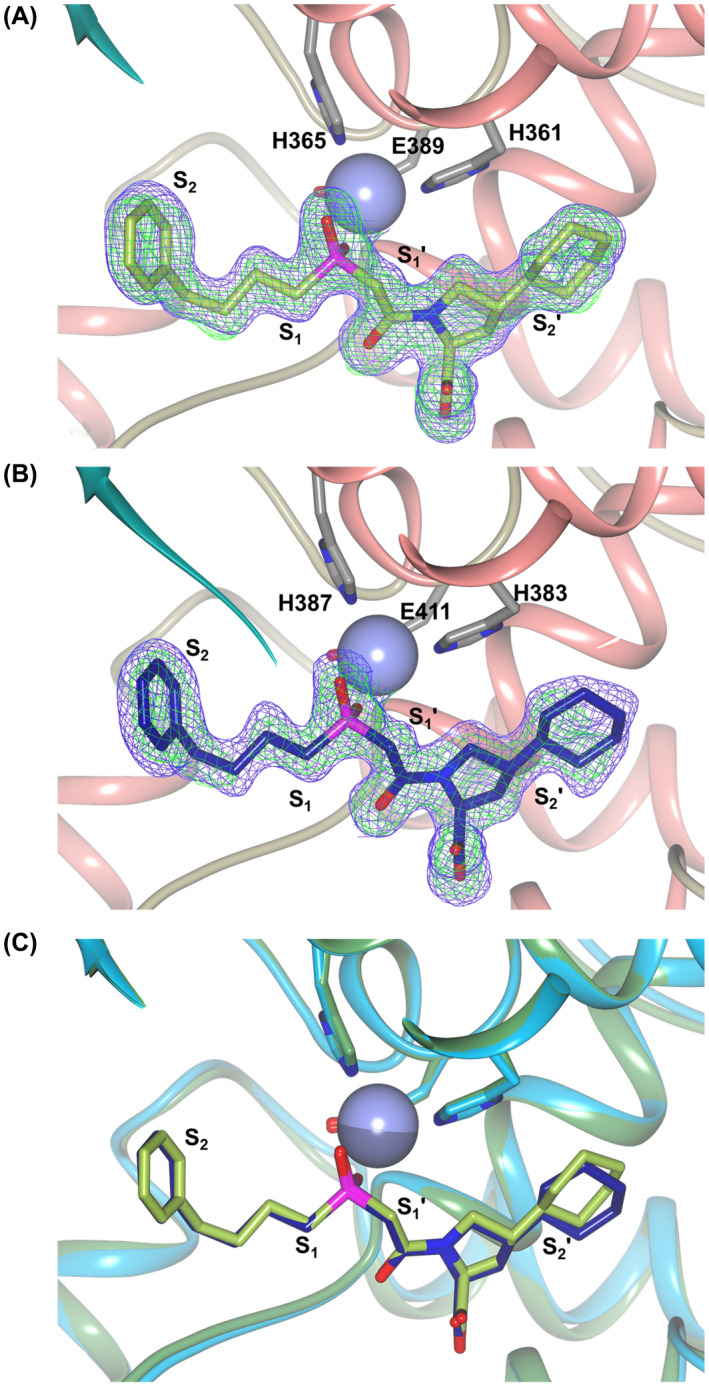
Fosinoprilat bound in the active sites of nACE and cACE. Schematic representations of (A) nACE‐fosinoprilat and (B) cACE‐fosinoprilat complexes showing the inhibitors bound to the active site zinc ion (lilac sphere) with final 2mFo‐DFc (blue, contoured at 1σ level) and the omit mFo‐DFc (green, contoured at 3σ level) electron density maps overlaid. Fosinoprilat molecules are shown as green (nACE) and blue (cACE) sticks, with helices, β‐strands and loops coloured in rose, dark cyan and tan, respectively. The nonprime and prime subsites are indicated. (C) Overlay of the nACE (green) and cACE (blue) fosinoprilat binding sites. Zinc ions are shown as spheres coloured light (nACE) and dark (cACE) lilac, with the nonprime and prime subsites labelled. Figure was generated using ccp4mg [66].

The fosinoprilat complex structures were determined to high resolutions of 1.75 Å for nACE (*R*_work_ of 0.182 and *R*_free_ of 0.219) and 1.85 Å for cACE (*R*_work_ of 0.185 and *R*_free_ of 0.209), the data processing and refinement statistics are shown in Table 1. As commonly observed for ACE domain structures, the nACE crystal had two molecules in the asymmetric unit (ASU) with a P1 space group, whereas the cACE crystal ASU had 1 molecule and the P2_1_2_1_2_1_ space group. Both showed the typical mostly α‐helical ellipsoid ACE structure in the closed conformation (Fig. 2), which when superposed were clearly very similar with a RMSD value of 0.90 Å (calculated for 556 Cα atoms). The ellipsoid is formed from two lobes that open to allow access to the active site [44], with the N‐terminal 100 residues forming the ‘lid‐like’ region that is more flexible and may control substrate access [7]. Examination of the mFo‐DFc omit maps revealed clear, unambiguous electron density for fosinoprilat occupying the S_2_, S_1_, S_1_′ and S_2_′ subsites in both the nACE and cACE complex structures (Fig. 3A,B).

**Table 1:** X‐ray data collection and refinement statistics. Data from the inner shell are shown in square brackets, overall data are unbracketed, and data from the outer shell are shown in rounded brackets, respectively.

	nACE‐fosinoprilat	cACE‐fosinoprilat
Resolution (Å)	[73.29–9.59], (1.78–1.75)	[134.69–9.06], (1.89–1.85)
Space group	P1	P2_1_ 2_1_ 2_1_
Cell dimensions (*a*, *b*, *c*) and angles (α, β, γ)	72.6, 77.8, 81.6 Å 88.9°, 64.6°, 74.8°	56.6, 86.0, 134.7 Å 90.0°, 90.0°, 90.0°
Molecules per asymmetric unit	2	1
Total/unique reflections	1 077 762/152 059	742 644/56 970
Completeness (%)	[99.5], 97.4, (94.2)	[99.9], 100.0, (100.0)
*R*_merge_	[0.033], 0.135, (2.252)	[0.102], 0.341, (3.144)
*R*_pim_	[0.013], 0.054, (0.952)	[0.032], 0.098, (0.903)
<*I*/σ*I*>	[35.0], 8.2, (0.8)	[13.9], 5.2, (0.7)
CC_1/2_	[0.999], 0.998, (0.438)	[0.992], 0.990, (0.327)
Multiplicity	[7.2], 7.1, (6.5)	[11.3], 13.0, (13.1)
Refinement statistics		
*R*_work_/*R*_free_	0.172/0.200	0.174/0.213
*R*_msd_ of bond lengths (Å)	0.008	0.005
*R*_msd_ of bond angles (°)	0.865	0.700
Ramachandran statistics		
Favoured	98.5	98.8
Allowed	1.3	1.0
Outliers	0.2	0.2
Average *B*‐factors (Å^2^)		
Protein	34.13	25.18
Ligand	49.69	40.60
Water	37.62	32.42
Number of nonhydrogen atoms		
Protein	10 032	4738
Ligand	918	265
Water	717	595
PDB code	7Z6Z	7Z70

### Ligand binding interactions

**Fig. 4:**
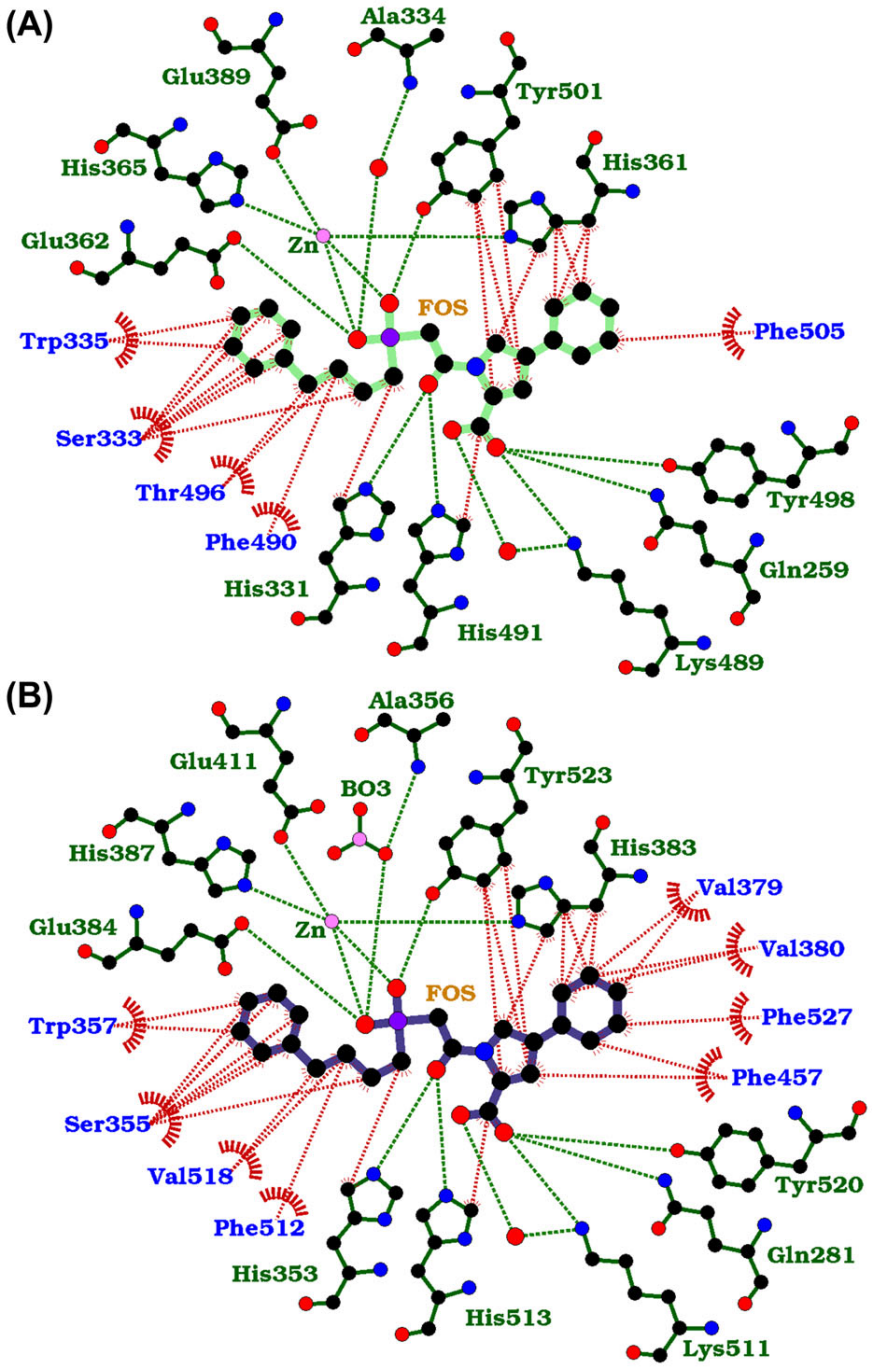
LigPlot representation of ACE‐fosinoprilat complexes. Comparison of (A) nACE and (B) cACE‐fosinoprilat binding site interactions. H‐bond/electrostatic and hydrophobic interactions are shown as green and red dashed lines, respectively, water molecules as red spheres and red, semicircular symbols depict residues solely involved in hydrophobic interactions. Figure was generated using ligplot+ [67].

The schematic representations of nACE‐ and cACE‐fosinoprilat complexes show the four‐carbon chain (P_1_) and phenyl group (P_2_) occupying the nonprime subsites and the peptide backbone mimic (P_1_′) and pyrrolidine‐cyclohexane group (P_2_′) occupying the prime subsites (Fig. 3A,B). Detailed analysis of both structures shows an extensive network of interactions in all the S_2_, S_1_, S_1_′ and S_2_′ subsites (Fig. 4), with all hydrogen‐bonding and electrostatic interactions with fosinoprilat being conserved between nACE and cACE. These are comprised of zinc binding from the phosphinic group, which additionally interacts via one oxygen with nACE‐Tyr501/cACE‐Tyr523 and the second oxygen with nACE‐Glu362/cACE‐Glu384. This second oxygen also has an indirect interaction with the backbone nitrogen of nACE‐Ala334/cACE‐Ala356, which is conserved, but mediated by a water molecule in nACE and a borate ion from the crystallisation conditions in cACE. The P_1_′ backbone oxygen forms hydrogen bonds with nACE‐His331/cACE‐His‐353 and nACE‐His491/cACE‐His513, while the terminal P_2_′ carboxylate group interacts directly with nACE‐Gln259/cACE‐Gln281, nACE‐Lys489/cACE‐Lys511 and nACE‐Tyr498/cACE‐Tyr520 and a water‐mediated interaction with nACE‐Lys489/cACE‐Lys511.

Most hydrophobic interactions are also conserved in both nACE‐ and cACE‐fosinoprilat complex structures. The P_1_ carbon chain and P_2_ phenyl group extends along a hydrophobic patch consisting of nACE‐His331/cACE‐His353, nACE‐Ser333/cACE‐Ser355, nACE‐Trp335/cACE‐Trp357, nACE‐Phe490/cACE‐Phe512 and nACE‐Thr496/cACE‐Val518. The P_2_ phenyl ring is also stabilised by nACE‐PEG/cACE‐ethylene glycol molecules from the crystallisation components; however, these molecules are not physiologically relevant so would not contribute to the *in vivo* affinity and domain selectivity of fosinoprilat. The P_2_′ backbone carbons interact with nACE‐His491/cACE‐His513 and nACE‐Tyr501/cACE‐Tyr523, while its pyrrolidine sidechain ring forms hydrophobic interactions with nACE‐His361/cACE‐His383, nACE‐Tyr501/cACE‐Tyr523 and at longer range with nACE‐Phe435/cACE‐Phe457.

An overlay of the nACE‐ and cACE‐fosinoprilat binding sites show that the P_2_′ sidechain cyclohexane ring is the only part of the inhibitor that has a significantly different orientation and position, which includes the ring being rotated about the pyrrolidine–cyclohexane bond (Fig. 3C). The following nACE and cACE residues both form hydrophobic interactions with the cyclohexane ring, namely nACE‐Ser357/cACE‐Val379, nACE‐Thr358/cACE‐Val380, nACE‐His361/cACE‐His383, nACE‐Phe505/cACE‐Phe527 and at longer range with nACE‐Phe435/cACE‐Phe457, but the relative distances vary between nACE and cACE. The details of these differences and how they relate to the affinity of fosinoprilat for nACE and cACE are discussed below.

## Discussion

### Comparison between nACE‐ and cACE‐fosinoprilat binding

**Fig. 5:**
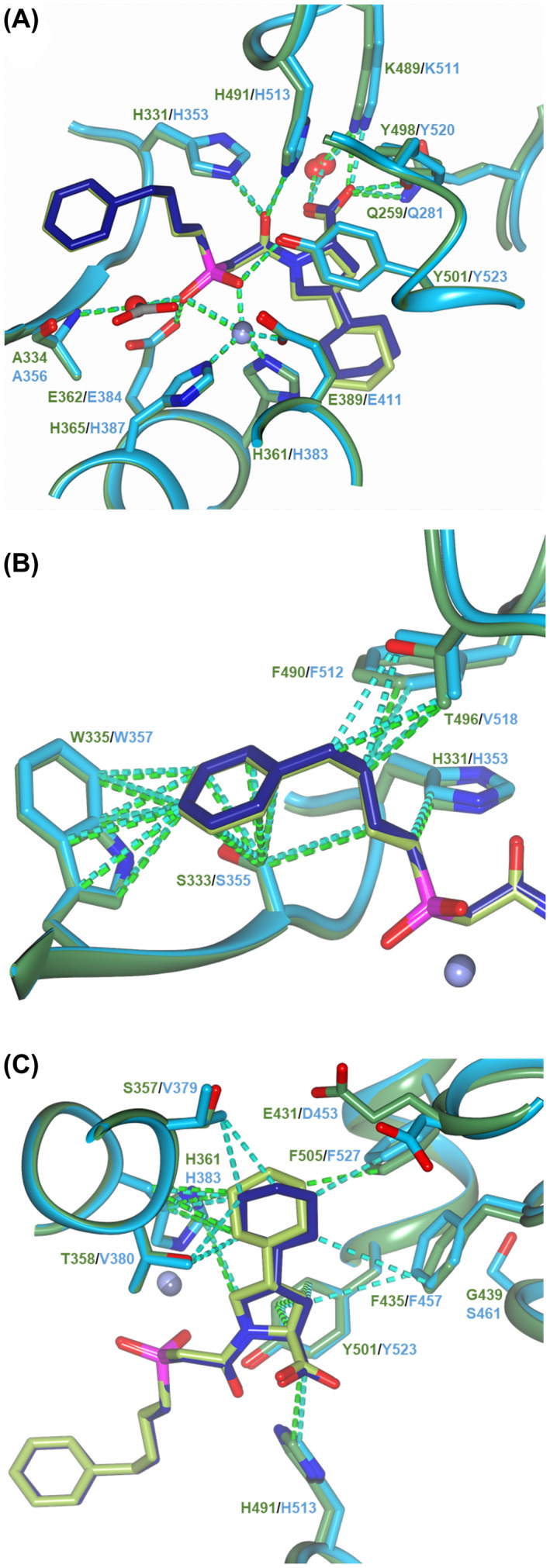
Schematic representations of the binding site interactions in ACE‐Fosinoprilat complexes. Overlays of nACE‐ and cACE‐fosinoprilat complex structures showing (A) electrostatic bonds and (B, C) nonprime and prime, respectively, subsite hydrophobic interactions. nACE and cACE are shown in green and blue, respectively, with interactions and labelling colour coordinated. Zinc ions are shown as spheres coloured light (nACE) and dark (cACE) lilac. Figure was generated using ccp4mg [66].

Fosinoprilat has an extremely high affinity for both nACE and cACE, as evidenced by the nanomolar *K*_*i*_ values; thus, we would anticipate numerous binding interactions throughout the prime and nonprime subsites. Indeed, the complex structures described above show this to be the case with the full length of the fosinoprilat molecules interacting with the ACE domains (Fig. 4). However, the kinetic studies showed that fosinoprilat has a 27.4‐fold selectivity for cACE over nACE. While all the hydrogen bond and electrostatic interactions with fosinoprilat are conserved (Figs 4 and 5A), the structures highlight differences in the hydrophobic interactions between the ACE domains that are consistent with changes in affinity.

Most of the S_1_ and S_2_ subsite hydrophobic patch is conserved between nACE and cACE (Figs 4 and 5B). However, a single mutation of nACE‐Thr496 to cACE‐Val518 changes the hydrophobicity of the S_1_ subsite, and while nACE‐Thr496 still provides hydrophobic interactions for the P_1_ 4‐carbon chain of fosinoprilat, it is not as optimal an environment as the S_1_ subsite in cACE. While this change in the S_1_ subsite could make a minor contribution to the C‐selectivity, the variation in environment for, and orientation of the P_2_′ sidechain is likely to have a greater effect (Figs 4 and 5C). Firstly, the OG1 atom of nACE‐Thr358 points directly towards the P_2_′ cyclohexane ring, whereas the equivalent cACE‐Val380 provides more hydrophobic interactions. Secondly, while the OG atom of nACE‐Ser357 points away from fosinoprilat, the corresponding cACE‐Val379 is more bulky and provides a closer hydrophobic patch to interact with the P_2_′ cyclohexane ring. nACE‐Glu431 shows some flexibility in the electron density maps and variation between the two molecules of the ASU. This allows one of the carboxylate oxygens to come within 4.4 Å of the P_2_′ sidechain which will lower the hydrophobicity of the S_2_′ subsite in nACE. In comparison, this residue in cACE is Asp453, which points away and is more distant from the bound fosinoprilat. Lastly, as previously observed [45], there is a small shift of cACE‐Phe457 towards the inhibitor molecule compared to the corresponding nACE‐Phe435 that is caused by the nACE‐Gly439/cACE‐Ser461 substitution (Fig. 5C). This is only likely to improve the hydrophobic environment by a small amount, but in combination with the other S_2_′ subsite changes described, it allows rotation of the P_2_′ cyclohexane ring resulting in overall more favourable hydrophobic interactions.

These results are in agreement with molecular docking studies [46] whereby the cyclohexane ring was oriented perpendicular to the pyrrolidine ring in nACE and parallel in cACE. Additionally, they suggested that the unique cACE‐Val379 and cACE‐Val380 residues would confer C‐domain selectivity via favourable hydrophobic interactions with the cyclohexane ring. Finally, the molecular docking studies predicted a favourable cation‐π interaction between the P_2_ phenyl group and the unique nACE‐Asn494 side chain; however in the structures presented here, this side chain was oriented in the opposite direction to the phenyl group.

Previous work has shown that ACE domain selectivity is not only dependant on the residues interacting with the inhibitor, but additionally by nonconserved residues located away from the binding site that are implicated in ACE opening and closing by interaction between subdomains and effects on hinge motion [44, 47, 48]. Therefore, the differences between nACE and cACE described above may not be solely responsible for the 27.4 fold C‐selectivity of fosinoprilat.

### Fosinoprilat binding mode in comparison with other domain‐specific inhibitors

In order to rationalise the extent of fosinoprilat C‐domain selectivity, comparisons were made between the interactions of the structures presented here and those of well‐characterised, domain‐selective ACE inhibitors. RXPA380 (Fig. 1B) is a highly C‐domain‐specific inhibitor whose *K*_i_ is 3000‐fold lower for cACE than nACE [35]. The main features responsible for this selectivity are the aromatic interaction between cACE‐Phe391 and the P_2_ phenyl moiety (the equivalent nACE‐Tyr369 is less hydrophobic and would cause steric hindrance), the hydrophobic interactions between cACE‐Val379 and cACE‐Val380 and the P_2_′ tryptophan group, and the additional space in the cACE S_2_′ pocket providing a more favourable environment for the large tryptophan group [35]. Fosinoprilat shows similar increased hydrophobic interactions in the S_2_′ subsite of cACE compared to nACE (Figs 4 and 5C), but lacks close proximity to nACE‐Tyr369/cACE‐Phe391.

Another factor determined from the study of the highly C‐domain‐selective inhibitor kAW [49] to confer selectivity was the favourable displacement of water molecules around cACE‐Val518 upon binding of a highly hydrophobic group in the nonprime subsites. In nACE, this causes the enthalpically unfavourable displacement of hydrogen‐bonded water molecules from the polar Thr496 side chain. In the ACE–fosinoprilat complex structures, the P_1_/P_2_ groups bind in the same close proximity to nACE‐Thr496/cACE‐Val518 as observed for kAW and could have a similar effect on water molecule binding. Indeed, there was no additional electron density close to nACE‐Thr496 or cACE‐Val518 that would indicate a bound water molecule, and therefore, this may be an additional contribution to the C‐selectivity of fosinoprilat. cACE‐Glu376 is also thought to confer selectivity via a water‐mediated hydrogen bond with the P_2_′ tryptophan group of kAW, although this was not observed in the cACE‐RXPA380 structure. Fosinoprilat does not have a hydrogen‐bond competent group in its P_2_′ moiety that could replicate this interaction. A final factor that could contribute to C‐domain specificity identified with the modest C‐domain selectivity of sampatrilat and lisinopril is the formation of an electrostatic interaction between an inhibitor P_1_/P_2_ lysine group and cACE‐Glu162, which is unable to form with the shorter nACE‐Asp140 side chain [6, 50]. This is not replicated with the domain selectivity of fosinoprilat due to its highly hydrophobic P_1_/P_2_ groups.

The interactions that confer high N‐domain selectivity are best exemplified by RXP407 [34], a phosphinic inhibitor with approximately 200‐fold selectivity for the N‐domain (Fig. 1A). The origins of this selectivity are the direct hydrogen bonds between the P_2_ aspartate and the unique Tyr369 and Arg381 residues within the nACE S_2_ subsite [36]. Tyr369 is substituted for the nonpolar Phe391 in cACE and Arg381 is substituted for Glu403, which has a shorter side chain so does not reach far enough into the S_2_ pocket to form hydrogen‐bonding interactions. These same residues were also shown to confer the N‐selectivity of compounds 33RE and a novel diprolyl inhibitor [27, 51]. In the case of fosinoprilat, not only are hydrogen‐bonding interactions with these residues not possible due to the lack of a P_2_ hydrogen‐bonding partner, but the fosinoprilat P_2_ substituent is also too small for it to come into close enough proximity to form meaningful interactions. The observation that either one or both of these interactions are present with three highly N‐domain‐selective inhibitors demonstrates their key importance, and therefore, the inability of these interactions to form between nACE and fosinoprilat provides a clear reason for why it is not N‐domain‐selective.

This comparison with other domain‐selective ACE inhibitors provides a strong argument for why the C‐selectivity of fosinoprilat is weak, in only having some of the functionality shown by other highly selective cACE inhibitors and none of the features of nACE selective inhibitors. To increase the C‐selectivity of fosinoprilat the P_2_′ group could be modified to introduce an aromatic interaction with cACE‐Phe391, which would also cause steric hindrance with nACE‐Tyr369. To further optimise the P_2_′ group a hydrogen‐bond competent group could be added to interact with cACE‐Glu376. The other end of the fosinoprilat molecule could also be modified to add a moiety to the P_2_ group to introduce an interaction with cACE‐Glu162, but care is needed to retain the hydrophobic environment around nACE‐Thr496/cACE‐Val518.

### Comparison of sACE‐ and AnoACE2‐fosinoprilat complexes

The only other ACE‐fosinoprilat crystal structure available is the complex with an *Anopheles gambiae* homologue, AnoACE2 [52]. *A. gambiae* is a species of mosquito that acts as a malarial vector, and fosinoprilat has been demonstrated to be lethal to developing larvae [53]. Comparisons between AnoACE2 and the human ACE structures presented here will identify whether regions that confer domain specificity between nACE and cACE differ between species and may suggest residues to target in AnoACE2 that could improve the efficacy of ACE inhibitors as a new class of insecticides while having low affinity for human ACE.

**Fig. 6:**
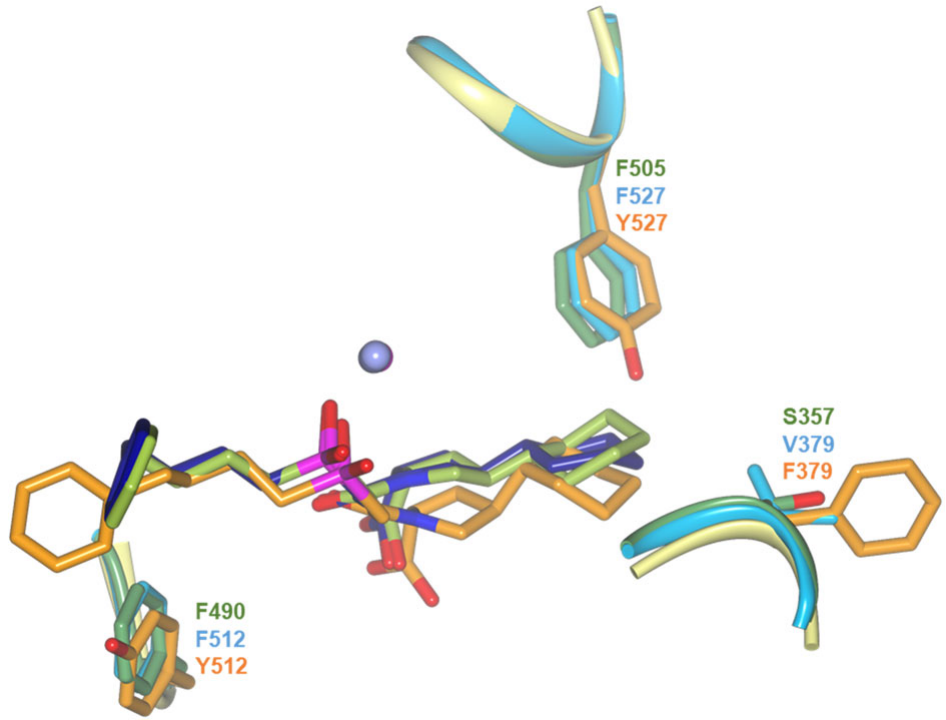
Overlay of fosinoprilat binding in nACE, cACE and AnoACE2 complexes. Comparison of the fosinoprilat conformations with nACE, cACE and AnoACE2 structures shown in green, blue and orange, respectively. Residues that are unique in AnoACE2 are highlighted. Zinc is shown as a lilac sphere. Figure was generated using ccp4mg [66].

A comparison of the binding sites of these domains and homologue indicate that AnoACE2 is more closely related to cACE than nACE. However, an overlay of the nACE, cACE and AnoACE2 fosinoprilat complex structures show that while the inhibitor molecules are orientated the same way and occupy the same subsites, there are significant shifts and some localised rotations of the AnoACE2 fosinoprilat molecule (Fig. 6). The binding site for ligand C‐terminal carboxylate group is conserved between ACE domains and homologues and varies very little between different complex structures. Therefore, it is unusual to see that the P_2_′ carboxylate of fosinoprilat in the AnoACE2 structure does not overlay well with the nACE and cACE complex structures. This results in suboptimal interactions with the usual C‐terminal carboxylate binding residues. The cause for this shift appears to be a mutation of nACE‐Phe505/cACE‐Phe527 to AnoACE2‐Tyr527. The OH atom of this tyrosine residue would not only sterically hinder the binding position of the fosinoprilat P_2_′ cyclohexane ring, but also reduce the hydrophobicity achieved with the phenylalanine residues of nACE and cACE. This results in the cyclohexane ring being pushed further down in the binding site and the rigid nature of fosinoprilat means there is a knock on effect to the rest of the molecule. This not only shifts the adjacent pyrrolidine ring, but also the carboxy terminus, the zinc binding phosphinic group and may also be the cause for the change in orientation of the P_1_ and P_2_ groups. Even though there is this shift, many of the nACE/cACE interactions with fosinoprilat are retained in AnoACE2, but there are some important differences. The hydrophobic interactions of cACE‐Val379 are reduced with AnoACE2‐Phe379 due to the increased distance from the P_2_′ cyclohexane ring. The phenylalanine ring points away from the bound inhibitor, likely due to there being insufficient space available to be orientated the opposite way when fosinoprilat is bound. However, it is conceivable that an inhibitor with a different P_2_′ might allow this Phe379 to rotate around providing strong interactions. Therefore, both Phe379 and Tyr527 of AnoACE2 are strong candidates to target for AnoACE2‐specific inhibitors.

The remaining significant differences in fosinoprilat binding in the AnoACE2 structure are found in the S_1_ and S_2_ subsites where the change in orientation of the P_1_ and P_2_ groups results in much reduced hydrophobic interactions even though most residues are conserved. This comparison highlights another difference, where nACE‐Phe590/cACE‐Phe512 is replaced by Tyr512 in AnoACE2. While this residue can provide some of the hydrophobic interactions seen in the nACE and cACE complexes, it is also a promising candidate to target in the design for AnoACE2‐specific inhibitors containing hydrogen‐bonding P_2_ groups.

Given the understanding of the key residues in nACE and cACE that interact with fosinoprilat, it may now be possible to modify/redesign fosinoprilat to exploit the unique residues of, and thereby increase its affinity for, AnoACE2. The analysis above indicates that the P_2_′ group could be modified to better fit in the S_2_′ subsite and target interactions with either or both Phe379 and Tyr527. In addition, the P_2_ group could be re‐designed such that there are strong interactions with Tyr512. This approach will maximise the specificity of AnoACE2 inhibitors that could be used as lead compounds for the development of novel insecticides.

## Conclusion

The high‐resolution structures of nACE and cACE in complex with fosinoprilat have allowed in depth analysis of their binding modes, with stronger hydrophobic interactions proposed as the main reasons for the observed C‐domain specificity. Comparison with other known inhibitors has indicated the lack of numerous key interactions proposed to confer high domain selectivity, thus providing a rationale for the weak selectivity of fosinoprilat. Finally, structural comparisons with the AnoACE2 structure have highlighted key residues that could be targeted for the development of novel insecticides.

## Materials and methods

### Enzymes and the inhibitor

Minimally glycosylated truncated N‐domain (N389) and truncated C‐domain (g13) (Ser‐1 to Pro‐633) human ACE proteins were generated by expression in cultured mammalian CHO cells and purified to homogeneity as described previously [36, 54]. Fosinoprilat was a gift from Bristol‐Myers Squibb (BMS, Princeton, NJ, USA).

### Kinetic characterisation of fosinoprilat binding

To determine fosinoprilat binding affinity, an assay using a modified *Z*‐phenyl‐alanyl‐histidyl‐leucine (Z‐FHL) (Bachem Ltd.) was performed [31]. Briefly, fosinoprilat was dissolved in distilled water and a serial dilution prepared in 100 mm potassium phosphate buffer, pH 8.3, containing 300 mm NaCl and 10 μm ZnSO_4_. Equal volumes of enzyme (4 nm) and fosinoprilat were mixed and incubated for 40 min at room temperature and 25 mL of the reaction aliquoted to a 96‐well plate in triplicate. Twenty‐five microliters of Z‐FHL at 1 mm was added to each well, incubated for 15 min at 37 °C and the remainder of the assay performed as described by Schwager et al. [55]. Initial reaction velocities were analysed using nonlinear regression in graphpad prism v.6.0 (San Diego, CA, USA) to obtain IC_50_ values from which *K*_i_ values were calculated using the Cheng–Prusoff equation [31, 56] (where [*S*] is the final substrate concentration of 0.5 mm Z‐FHL). IC_50_ were carried out in triplicate.

(1) $$K_{i}=\frac{{\mathrm{IC}}_{50}}{1+\frac{\left[S\right]}{K_{m}}}.$$

### X‐ray crystallography of nACE‐ and cACE‐fosinoprilat complexes

The ACE domains were pre‐incubated with fosinoprilat in a 4 : 1 v/v ratio of protein (8 mg·mL^−1^ G13‐cACE and 5 mg·mL^−1^ N389‐nACE in 50 mm Hepes, pH 7.5, 0.1 mm PMSF) and 20 mm inhibitor for 1 h (cACE on ice, nACE at room temperature). Co‐crystals were obtained with hanging drops of 1 μL of the protein‐inhibitor complex mixed with equal volume of reservoir solution (0.1 m MIB buffer pH 4.0, 5% glycerol and 15% PEG 3350 for cACE‐fosinoprilat and 30% PEG 500 MME/PEG 20000, 0.1 M Tris/Bicine pH 8.5 and 60 mm divalent cations, Molecular Dimensions [Rotherham, UK] morpheus A9 for nACE‐fosinoprilat). Both proteins were then incubated at 16 °C.

X‐ray diffraction data were collected on stations i03 (nACE) and i04 (cACE) at the Diamond Light Source (Didcot, UK). Crystals were kept at constant temperature (100 K) under the liquid jet during data collection. Images were collected using Eiger2 XE 16M detector (Dectris, Baden – Daettwil, Switzerland). Raw data images were indexed and integrated with dials [57] and then scaled using aimless [58] from the ccp4 suite [59]. Initial phases for the native structure were obtained by molecular replacement with phaser [60]. PDB codes 6F9V and 6F9T [61] were used as the search models for nACE and cACE, respectively. Further refinement was initially carried out using refmac5 [62] followed by phenix [63], with coot [64] used for rounds of manual model building. Fosinoprilat, zinc and chloride ions, purification/crystallisation buffer reagents and water molecules were added based on electron density in the *F*_o_ − *F*_c_ Fourier difference map. molprobity [65] was used to help validate the structures. Crystallographic data statistics are summarised in Table 1. All figures showing the crystal structures were generated using either ccp4mg [66], and schematic binding interactions are displayed using ligplot+ [67].

## Conflict of interest

The authors declare no conflict of interest.

## Author contributions

GEC performed crystallisation, collected high‐resolution X‐ray diffraction data, analysed the data and edited the manuscript. ECN performed crystallographic structure analysis and wrote the manuscript. SLUS carried out the expression and purification of nACE and cACE and the kinetic characterisation of fosinoprilat binding. REI provided fosinoprilat used in this study and edited the manuscript. EDS analysed the data and edited the manuscript. KRA supervised the study, analysed the data and edited the manuscript. All authors reviewed the manuscript.

### Peer review

The peer review history for this article is available at https://publons.com/publon/10.1111/febs.16543.

## Data Availability

The atomic coordinates and structure factors of nACE‐fosinoprilat and cACE‐fosinoprilat complexes have been deposited in the Protein Data Bank under accession codes 7Z6Z and 7Z70, respectively.
